# ^13^C-Enrichment of Urinary Uric Acid after l-[Ring-2-^13^C]Histidine Dose in Adult Humans

**DOI:** 10.3390/nu7010697

**Published:** 2015-01-20

**Authors:** Tsunenobu Tamura, Joseph E. Baggott

**Affiliations:** Department of Nutrition Sciences, University of Alabama at Birmingham, Birmingham, AL 35294, USA; E-Mail: marilynbaggott@att.net

**Keywords:** C_2_ enrichment, uric acid, folate metabolism, humans, purine nucleotide biosynthesis, [ring-2-^13^C]histidine

## Abstract

We determined whether ring-2 carbon of histidine is folate-dependently transferred to carbons 8 (C_8_) and/or 2 (C_2_) in urinary uric acid in humans. Two adults collected each urine void for four days. Aliquots of urine for the first day were used for baseline values; then the subjects ingested 0.7 g (3.3 mmol) of l-[ring-2-^13^C]histidine and collected urine for three experimental days. Aliquots were analyzed for percentage ^13^C-content at C_2_ and C_8_ by a liquid-chromatography-mass spectrometry method. Percentage enrichment was determined by subtracting time-of-day paired baseline percentage ^13^C-content from experimental percentage ^13^C-content for each void. C_2_ was predominantly ^13^C-enriched in the majority of voids. The percentage enrichments at C_2_ for two subjects were 0.14 (±0.028 [SEM], *n* = 26) and 0.18 (±0.049, *n* = 21), whereas at C_8_, they were 0.008 (±0.006) and −0.005 (±0.008), respectively. The mean C_2_-enrichments were significantly greater than zero (*p* < 0.01), whereas those of C_8_ were not (*p* > 0.2). The enrichment had a diurnal rhythm peaking in the morning. Our results may be useful in the estimation of the timing for the administration of drugs that interfere with purine nucleotide biosynthesis in the treatment of cancer and autoimmune disease.

## 1. Introduction

**Figure 1:**
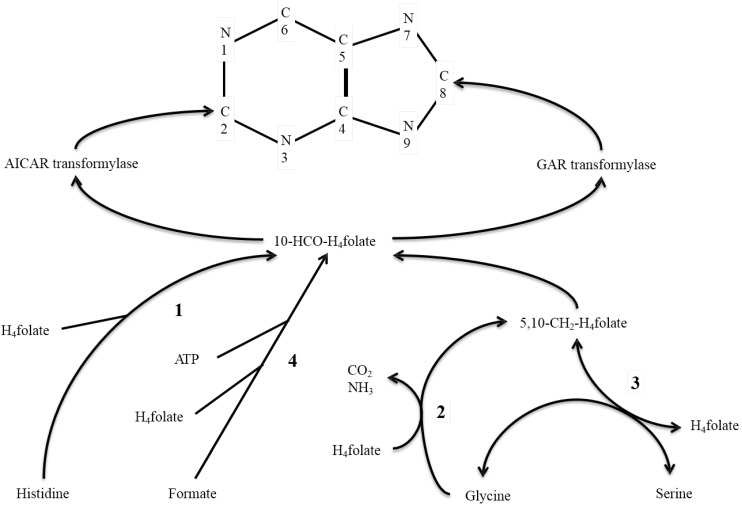
Metabolic pathway of 10-formyltetrahydrofolate (10-HCO-H_4_folate) formed from tetrahydrofolate (H_4_folate) and [ring 2-carbon]histidine, [3-carbon]serine, [2-carbon]glycine, and formate. Enzymes include: (1) 5-formiminotetrahydrofolate transferase-cyclodeaminase; (2) glycine-cleavage system; (3) serine hydroxylmethyltransferase; and (4) 10-HCO-H_4_folate synthetase. 10-HCO-H_4_folate is incorporated into the carbons 2 (C_2_) and 8 (C_8_) of the purine ring by AICAR and GAR transformylases, respectively.

Purine nucleotide biosynthesis *de novo* is an essential process in producing building blocks of DNA and RNA [1]. Two folate-dependent enzymes, glycinamide ribotide (GAR) and aminoimidazole carboxamide ribotide (AICAR) transformylases, are involved in the process for the transfer of one-carbon units into the positions 8 (C_8_) and 2 (C_2_) of the purine ring, respectively (Figure 1). Uric acid is the final catabolic product of purines in humans and is excreted in urine, and this catabolism does not alter the positions of carbons in the ring [1]. Using a liquid-chromatography-mass spectrometry (LC/MS/MS) method [2,3], it is possible to independently measure the ^13^C-enrichment at the C_2_ and C_8_ positions of urinary uric acid after an oral dose of ^13^C-labeled sources, which include formate, 2-glycine, 3-serine and ring-2-histidine. Among those one-carbon sources, only histidine is considered to be essential in humans [4].

In our previous studies, the ^13^C-enrichment independently at the C_2_ and C_8_ positions of urinary uric acid was measured after an oral dose of ^13^C-formate and [2-^13^C]-glycine, and these enriched mainly C_2_ and only C_8_ (+C_5_) of urinary uric acid, respectively [3]. In the present study, we determined whether l-[ring-2-^13^C]histidine furnishes a one-carbon to C_8_ and/or C_2_. To our knowledge, such a fundamental human study using the ring-2-histidine as a tracer has never been reported.

## 2. Subjects and Methods

This study was approved by the Institutional Review Board for Human Use at the University of Alabama at Birmingham (F060417002 approved in 2006), and written informed consent was obtained. For a total of four days, two healthy adult males collected and measured the volume of each urine void after each urine void was thoroughly mixed. The subjects were two healthy adults with no history of major illness, who were consuming normal diet without folic acid supplementation. Their serum and red-cell folate concentrations are within normal range. Serum vitamin B-12 and plasma pyridoxal 5′-phosphate concentrations were also normal, and their genotype of 5,10-methylenetetrahydrofolate reductase was the normal variant (677 CC). Aliquots of each void for the first 24-h period were used for analysis for baseline values. Subsequently, the subjects ingested 0.7 g (3.3 mmol) of l-histidine-[ring-2-^13^C]∙HCl∙H_2_O (98%, Cambridge Isotope Laboratories, Andover, MA, USA) with 50-mL water at 12:00–16:00 hour and continued collection of each urine void for three experimental days. Samples of all urine voids were analyzed for percentage ^13^C-content at C_2_ and C_8_ of urinary uric acid by a LC/MS/MS method [2,3]. Percentage enrichment was determined by subtracting time-of-day paired baseline percentage ^13^C-content from experimental percentage ^13^C-content for each void in both subjects. Significance of the mean percentage enrichment above zero was determined by the paired-*t*-test independently for C_2_ and C_8_ of urinary uric acid. Since values in urine voids reflect *in vivo* metabolic events hours earlier, and voids were not equally spaced in time, these data were not suitable for traditional circadian analysis. However, the large variation in the percentage ^13^C-enrichment at C_2_ suggested the use of the runs-above- and below-the-median test that assumes no mathematical model [5].

## 3. Results

**Figure 2:**
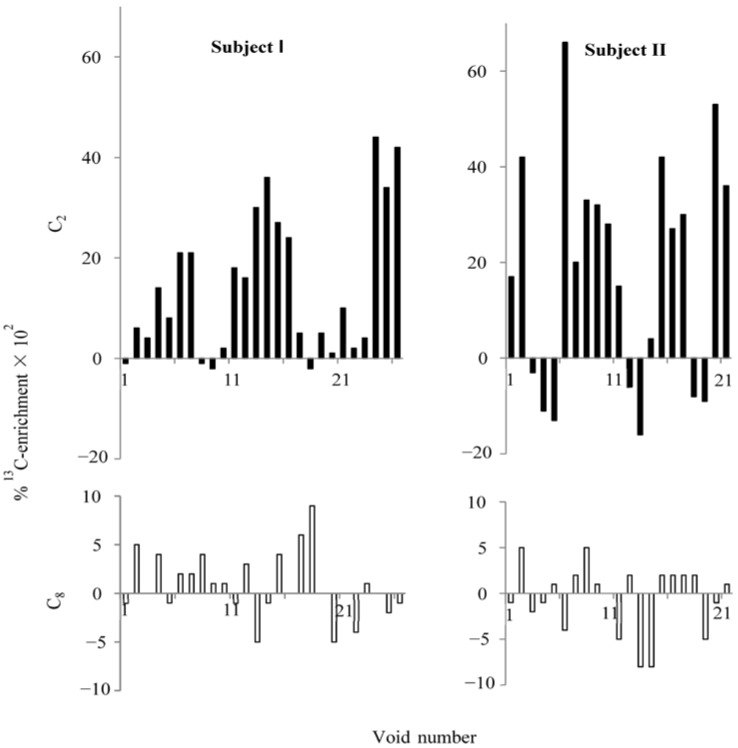
Percentage ^13^C-enrichment at C_2_ and C_8_ of uric acid in urine voids after ingesting 2-^13^C-histidine. Subjects I and II had 26 and 21 urine voids, respectively. Closed bars represent percentage ^13^C-enrichment at C_2_, and open bars represent percentage ^13^C-enrichment at C_8_. Note that the scales on the y-axes are different between C_2_ and C_8_.

After an oral ingestion of l-[ring-2-^13^C]histidine, C_2_ was predominantly ^13^C-enriched in the majority of urine voids collected for three experimental days (Figure 2). The mean (±SEM) percentage enrichments at C_2_ for two subjects (I and II) were 0.14 (±0.028, *n* = 26) and 0.18 (±0.049, *n* = 21), whereas at C_8_ they were 0.008 (±0.006) and -0.005 (±0.008), respectively. Mean C_2_-enrichments were significantly greater than zero (*p* < 0.01), whereas those of C_8_ were not (*p* > 0.2). There was no significant correlation between uric acid excretion and percentage ^13^C enrichment at C_2_ in each void, because about one third of the urine voids had little or no enrichment. Based on the previous study [3], any enrichment less than 0.004% was not considered significantly greater than zero. Subjects ingested histidine at different times, and voids are not spaced equally in time. Therefore, there is not a perfect overlap of the enrichments in Figure 2.

In our subjects I and II, the uric acid excretion was 9.1 and 9.8 mmol per three days, respectively. The uric acid content in each void varied from 8% to 16% of the total daily uric acid excretion during the period. The fraction of the dose of histidine excreted as uric acid was 0.39% and 0.54% in three days, respectively.

**Figure 3:**
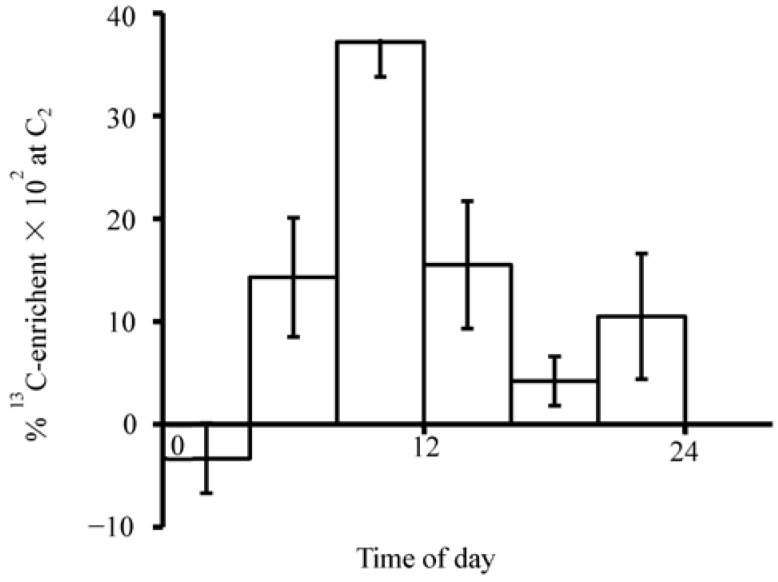
Diurnal changes in mean percentage ^13^C-enrichment at C_2_ after ingesting 2-^13^C-histidine. These are based on the data at six 4-h periods (Figure 2). Each column from left to right includes data from 6, 6, 12, 11, 6, and 6 urine voids. Vertical line represents SEM. In order to make the figure more compact, the top of this error bar (08:00–12:00) was cut off.

Diurnal rhythm was detected by the runs-above- and below-the-median test [5]. The results indicated eight runs each for subjects I and II (*p* < 0.01). This was less than expected indicating the presence of rhythmicity. As shown in Figure 3, the mean percentage ^13^C-enrichment at C_2_ during 8:00–12:00 h of 0.37 (±0.034, *n* = 12) was significantly higher than that during 20:00–24:00 h plus 00:00–04:00 h of 0.035 (±0.014, *n* = 12, *p* < 0.01).

## 4. Discussion

In the present study, we found that the C_2_ position of urinary uric acid was predominantly ^13^C-enriched in the majority of urine voids collected for three experimental days following an oral ingestion of l-[ring-2-^13^C]histidine. The mean enrichments at C_2_ for two subjects were 0.14% and 0.18%, whereas at C_8_ they were 0.008% and -0.005%, respectively. In our previous study, these two subjects participated as well and ingested [^13^C]formate and [2-^13^C]glycine, which enriched mainly C_2_ and only C_8_ (+ C_5_) of urinary uric acid, respectively [3]. We have now determined the locations of the incorporation of all one-carbon sources with the exception of serine. It is well established that [2-^13^C]glycine produces [3-^13^C]serine by the glycine-cleavage-system reactions coupled with ^13^C-exchange by serine hydroxylmethyltransferase [6,7]. Therefore, we postulate that [3-^13^C]serine also enriches principally C_8_. Matthews *et al.* [8] reported that 54% of plasma serine was biosynthesized from glycine in 60 hours in adult males. These findings suggest that [10-^13^C]formyltetrahydrofolate originating from [3-^13^C]serine is channeled to GAR transformylase in a folate-requiring-enzyme complex containing the trifunctional folate-metabolizing enzyme and serine hydroxymetyhyltransferase [9,10], whereas ring-2-histidine and formate furnish their one carbon predominantly to AICAR transformylase. Human GAR transformylase exists as a part of a large multifunctional protein which is extended and highly flexible [11] and could allow the trifunctional folate-metabolizing enzyme and serine hydroxymetyhyltransferase to form a complex as has been observed in chicken liver enzymes [9,10]. This complex would channel one carbon from 3-serine to C_8_. Therefore, human GAR transformylase multifunctional protein could be used as a platform to bind other enzymes as previously suggested [11].

In order to delineate possible mechanism(s) and implication of our finding of predominant enrichments at the C_2_ position after a l-[ring-2-^13^C]histidine load, it might be worth to note the following. Aminoimidazolecarboxamide (AICA), a metabolite of AICAR, is normally found in urine of healthy humans. Therefore, purine nucleotide biosynthesis is likely being blocked at this step to a certain extent at all times [3,12]. AICAR transformylase, not associated with a folate-requiring-enzyme complex that utilizes a non-essential amino acid, may be more sensitive than GAR transformylase to changes in folate-coenzyme pools and in the ability of folates to acquire a one carbon that is destined for C_2_. We postulate that GAR transformylase has a metabolic advantage over AICAR transformylase in acquiring a one carbon, because GAR transformylase precedes AICAR transformylase in purine nucleotide biosynthesis and utilizes the non-essential glycine and serine from diet as well as from their biosynthesis. In contrast, AICAR transformylase may have to depend on low *in vivo* pools of formate and the essential amino acid, histidine. Dietary histidine is required for protein biosynthesis and other homeostatic processes; therefore, it may seem metabolically wasteful to catabolize it to the non-essential glutamic acid via folate-dependent enzymes, formiminotransferase-cyclodeaminase [13]. However, if ring-2-histidine is important as a one-carbon source for C_2_, this seemingly wasteful catabolism of histidine may be essential. The catabolism of histidine must be important especially early in life, because the deficiency of formiminotransferase is manifested by growth retardation and neurological abnormalities [14]. In patients with this defect, more urinary AICA excretion is observed with and without an oral AICA load compared to healthy individuals [15]. This indicates that folate-dependent histidine catabolism to produce 10-formyltetrahydrofolate (formed from 5-formiminotetrahydrofolate) is apparently limiting the supply of a one carbon to AICAR transformylase. Furthermore, [ring-2-^14^C]-histidine was reported to label purines much more efficiently than serine or the methyl groups of choline in rats, clearly suggesting that this one carbon is destined for purine nucleotide biosynthesis rather than supplying the general one-carbon pool [16].

As to the importance of histidine in purine biosynthesis, there is a unique article in the literature. Cooperman and Lopez [17] reported that an oral histidine load alone in folate-deficient adult patients resulted in an 18-fold increase in reticulocyte formation in five days. The reticulocyte response is what would be expected if folic-acid therapy had been initiated. Therefore, it is likely that the excess histidine supplies its one carbon for DNA and RNA synthesis in the erythropoietic system; hence correcting the anemia due to folate deficiency in these patients. This could also suggest that the one carbon from ring-2-histidine is used in both purine and thymine nucleotide biosyntheses. However, the exact mechanism explaining these findings remains unknown. It may be that formiminotransferase-cyclodeaminase could be associated with AICAR transformylase in bone marrow favoring the incorporation of a one carbon from ring-2-histidine into C_2_. The association of histidine catabolism with purine nucleotide biosynthesis in humans has largely been neglected, and further research in this area is warranted.

The ^13^C-enrichment at C_2_ after the doses of histidine or formate [11] had a diurnal rhythm in humans, which may reflect diurnal changes in AICAR, GAR, folate, histidine and formate pools as well as AICAR transformylase activity. In addition, the size of purine nucleotide pool may regulate this enrichment [18]. Since the urine voids reflect metabolic events hours earlier, the diurnal rhythm may indicate more active metabolic use of histidine and formate for purine nucleotide biosynthesis during the night-time than daylight hours resulting in enhanced C_2_ enrichment of uric acid in the morning. Our results may provide important information in deciding the timing for the administration of drugs that interfere with purine nucleotide biosynthesis (e.g., antifolates) in the treatment of cancer and autoimmune disease. In fact, it has been established that methotrexate efficacy for certain cancers is increased when the drug is given during the evening and night hours [19,20].

A limitation of the study is that the number of subjects in this study is only 2. Therefore, our results should be considered preliminary. However, the use of the specific l-[ring-2-^13^C]histidine tracer and the specific method to independently determine the ^13^C-enrichment by LC/MS/MS yielded consistent results in both subjects. We used 3.3 mmol of l-[ring-2-^13^C]histidine as a tracer, and this amount was close to the estimated daily dietary intake of 4.5 mmol [21]. Therefore, we believe that our data represent the typical metabolism of histidine and purine in the general adult population.

## 5. Conclusions

Our findings presented here can be summarized as follows: (a) C_2_ of urinary uric acid was predominantly ^13^C-enriched after a l-[ring-2-^13^C]histidine dose; (b) this enrichment showed a diurnal rhythm; and (c) folate-dependent histidine catabolism to glutamic acid may be essential for purine nucleotide biosynthesis. These results together with those in our previous study [3] are the first to document the independent detection of ^13^C-enrichment at C_2_ and C_8_ of urinary uric acid after ingestion of ^13^C-labeled formate, 2-glycine and ring-2-histidine in the same individuals. It is important to stress that the roles of folate coenzymes and one-carbon sources in purine nucleotide biosynthesis *de novo* described in general textbooks, have been obtained in uricotelic animals and microorganisms, where it was found or assumed that one-carbon sources from its precursors are equally incorporated into C_2_ and C_8_ of the purine ring. However, our results indicate that the above findings cannot be extrapolated to human metabolism. Our non-invasive method using ^13^C-labeled tracers and the LC/MS/MS method should lead to a better understanding of the purine biosynthesis in future human studies. Although our results represent a small sample size and should be considered preliminary, we hope that our findings stimulate the interest of researchers in the area of histidine metabolism and purine nucleotide biosynthesis in humans.
